# Cell-Based Fabrication of Organic/Inorganic Composite Gel Material

**DOI:** 10.3390/ma4010327

**Published:** 2011-01-24

**Authors:** Takuya Matsumoto, Ami Mizuno, Miki Kashiwagi, Shin-suke Yoshida, Jun-ichi Sasaki, Takayoshi Nakano

**Affiliations:** 1 Department of Oromaxillofacial Regeneration, Osaka University, 1-8 Yamada-oka, Suita, Osaka 565-0871, Japan; 2 Division of Materials & Manufacturing Science, Osaka University, Suita, Osaka 565-0871, Japan

**Keywords:** hydrogel, organic/inorganic composite, 3D, biomimetic material

## Abstract

Biomaterials containing components similar to the native biological tissue would have benefits as an implantable scaffold material. To obtain such biomimetic materials, cells may be great contributors because of their crucial roles in synthetic organics. In addition, the synthesized organics—especially those derived from osteogenic differentiated cells—become a place where mineral crystals nucleate and grow even *in vitro*. Therefore, to fabricate an organic/inorganic composite material, which is similar to the biological osteoid tissue, bone marrow derived mesenchymal stem cells (BMSCs) were cultured in a 3D fibrin gel in this study. BMSCs secreted bone-related proteins that enhanced the biomineralization within the gel when the cells were cultured with an osteogenic differentiation medium. The compositions of both synthesized matrices and precipitated minerals in the obtained materials altered depending on the cell culture period. The mineral obtained in the 3D gel showed low crystalline hydroxyapatite. The composite materials also showed excellent osteoconductivity with new bone formation when implanted in mice tibiae. Thus, we demonstrated the contributions of cells for fabricating implantable organic/inorganic composite gel materials and a method for controlling the material composition in the gel. This cell-based material fabrication method would be a novel method to fabricate organic/inorganic composite biomimetic materials for bone tissue engineering.

## 1. Introduction

Bone tissue has critical roles in supporting body weight, enabling motility, and protecting important organs [1,2]. Bone tissue also works as a reservoir for inorganic ions, such as calcium and phosphorus, and supplies a place for hematopoiesis to occur [3]. For bone defects caused by regional segmentation for tumor extractions, necrosis due to X-ray radiation, or injury [4,5], the bones are reconstructed using biomaterials, including titanium or sintered ceramic materials, to recover the bone functions [6,7]. This bone reconstruction supports body motility and rebuilds the morphology of the original tissue. However, metals or sintered ceramic biomaterials have limited abilities for generating more complicated bone functions such as supplying calcium and phosphorus or hematopoiesis. Therefore, bone tissue regeneration by combining biomaterials and biologic materials (e.g., cells and soluble factors) has been investigated in the past few decades [8,9,10]. For example, proteins, sugars, and bioceramics have been evaluated as functional carriers for cells and/or soluble factors and have significantly enhanced bone regeneration [11,12]. However, these biomaterials still have limited approval for clinical use because the natural polymers used in these biomaterials (e.g., type I collagen, hyaluronic acid) are often derived from domesticated animals. Therefore, biomaterials derived from autologous sources alone would have tremendous advantages.

On the other hand, integrating implanted biomaterials with host hard tissues is also considered a challenging issue in bone tissue engineering [13]. For this integration, fabrication of implantable materials that contain both organics and minerals similar to the components of biological osteoid tissue might be effective, because naturally derived organics generally have higher affinity to host cell and tissue. However, native bony tissue contains the huge kinds of organic molecules derived from cell addition to apatite minerals. Hence, culturing cells in a three-dimensional (3D) hydrogel material *in vitro* would be an effective approach to fabricate biomimetic osteoid-like materials, because cells themselves secret osteoid proteins that enhance the mineral precipitations.

Fibrin is a fibrous protein that forms fibrin clots to prevent further bleeding from an injured blood vessel. Because fibrinogen and thrombin, the source of fibrin, can be obtained from the peripheral blood of any individual [14], fibrin gel is one of the ideal biomaterials for tissue engineering purpose when applied to one’s self. Bone marrow derived mesenchymal stem cells (BMSCs) can differentiate into osteoblasts and secret a number of bone-related matrix proteins when they are cultured with a special osteogenic medium [15]. Moreover, the secreted proteins supply the location for mineral crystals to nucleate and grow [16].

Here, we hypothesized that culturing BMSCs in 3D fibrin gel would form organic/inorganic composite materials containing cells, bone matrix proteins, and minerals that are similar to biological osteoid tissue. Moreover, the matrix and mineral composition in the 3D constructs would be controlled by the cell culture conditions. To test these hypotheses, BMSCs were cultured in 3D fibrin gel for different culture periods (14–42 days) in floating culture condition. The chemical properties of the obtained 3D constructs were then investigated in this study.

## 2. Results and Discussion

### 2.1. BMSCs Culture in 3D Fibrin Gel

**Figure 1:**
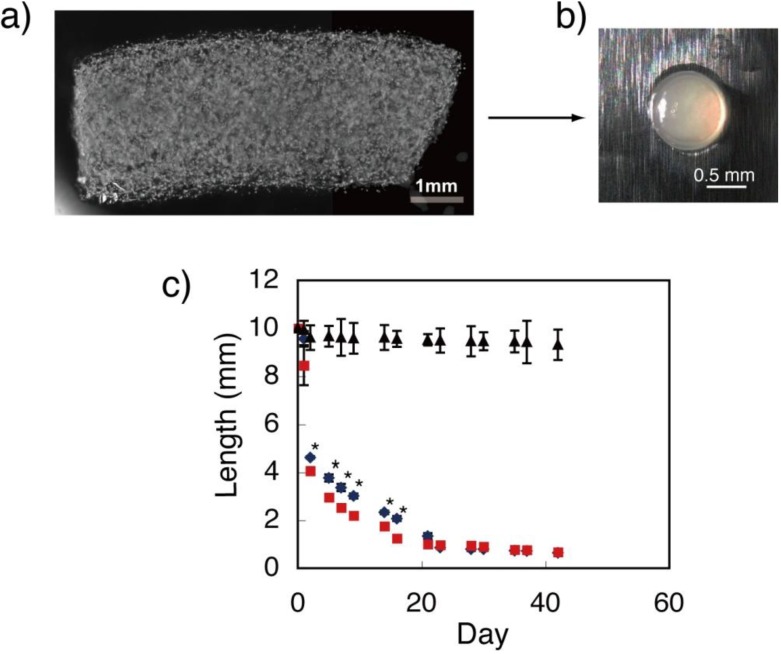
(**a**) Fibrin gel formed in silicone mold showing cylindrical morphology at initial stage; (**b**) Gel containing cells changing to spheroidal morphology during culture; (**c**) Gel size in long axis decreased over the culture period. Gel containing 8.0 × 10^4^ cells/mL (■) showed a greater decrease in gel size than that containing 2.0 × 10^4^ cells/mL (◆). Size of gel without cells (▲) slightly decreased during culture period. (* indicates significant difference (p < 0.01) between ■ and ◆).

Employing and culturing cells in 3D hydrogel would be a new method to synthesize organic/inorganic composite material that mimics biologically immature osteoid tissue. To address this goal, BMSCs were cultured in 3D fibrin gel with osteogenic differentiation medium. Fibrin gel containing cells that was formed in a silicone mold was initially cylinder-shaped but became spheroidal during the culturing (Figure 1). The gel size decreased dramatically when cells were contained in the gel (<90%: Length contraction). However, the gel size only decreased slightly when cells were not contained in the gel (<7%: Length contraction). These results suggest that gel contraction was caused by the cell traction force in the gel [17,18]. To confirm this, the size alterations of gels containing different cell numbers were investigated. Consequently, the gel containing 8.0 × 10^4^ cells/mL showed a greater decrease in size than the gel containing 2.0 × 10^4^ cells/mL by day 23 of culture (Figure 1). After the gels decreased to approximately 0.9 mm in diameter (at day 23), no significant difference of size was observed in further culture periods for each condition. Thus, the number of cells cultured in the gel would be the important factor of gel contraction.

### 2.2. Deposition and Distribution of Matrix and Minerals in Fibrin Gel

**Figure 2:**
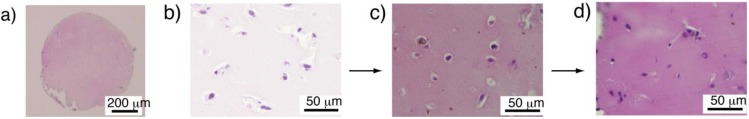
(**a**) Representative image of Hematoxylin and eosin (HE)-stained 3D construct (day 35); (**b–d**) Pink color indicating matrix protein deposition darkened over the cell culture period (b: day 28, c: day 35, d: day 42).

**Figure 3:**
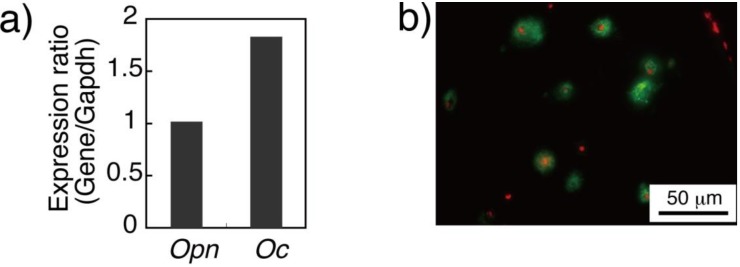
(**a**) Osteogenic gene expressions in BMSCs at day 14. Value of gene expression of osteopontin (Opn) and osteocalcin (Oc) was normalized to that of Gapdh; (**b**) Immunofluorescent staining of type I collagen indicated protein localization surrounding cells.

Hematoxylin and eosin (HE) staining of the fibrin gel containing BMSCs showed that the matrix distribution, indicated by eosin pink, extended in accordance with the cell culture period in the gel. In addition, the pink darkened in accordance with the cell culture period, indicating the increase in matrix protein deposition (Figure 2). Dark pink could especially be found around the cells, indicating that the stained matrix was derived from the cells. In osteogenesis, the osteoblastic cells secrete type I collagen and numerous non-collagenous proteins, including osteopontin, osteocalcin, and bone sialoproteins [19]. These non-collagenous proteins have critical roles in guiding biomineralization [20]. Real time RT-PCR data indicated sufficient gene expression of osteopontin and osteocalcin in the cells cultured in the fibrin gel. Type I collagen expression was also detected in the gel through immunofluorescent staining (Figure 3). Thus, it was confirmed that both collagen and non-collagenous proteins, found in native osteoid tissue, were contained in the 3D hydrogel fabricated in this study.

**Figure 4:**
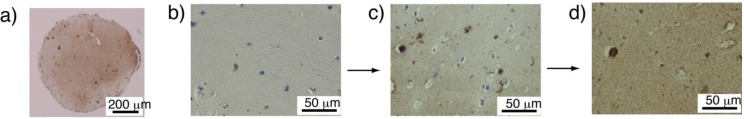
(**a**) Representative image of von Kossa-stained 3D construct (day 35); (**b–d**) Brown color indicating mineral deposition darkened over the cell culture period (b: day 28, c: day 35, d: day 42).

**Figure 5:**
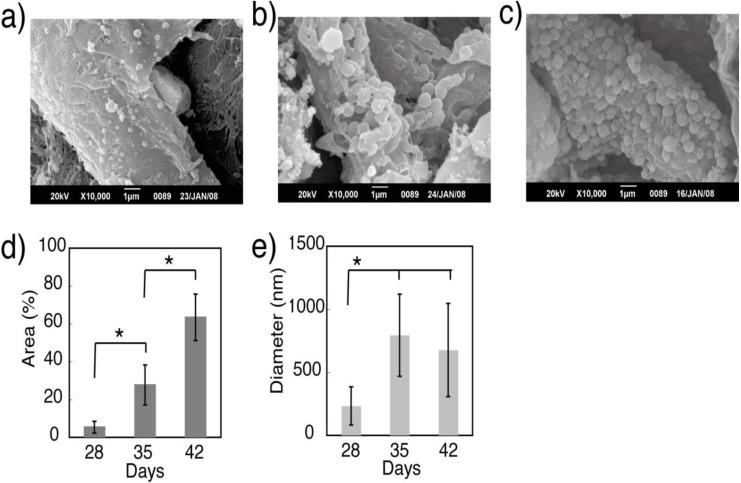
(**a–c**) Scanning electron micrograph (SEM) images indicating that mineralized matrix vesicles on cell membranes increased over the duration of the culture period (a; day 28, b; day 35, c; day 42); (**d**) Area ratio occupied by mineralized vesicles on single cell; (**e**) Alteration of mineralized vesicle mean size. For (d) and (e), * indicates significant difference (P < 0.01).

**Figure 6:**
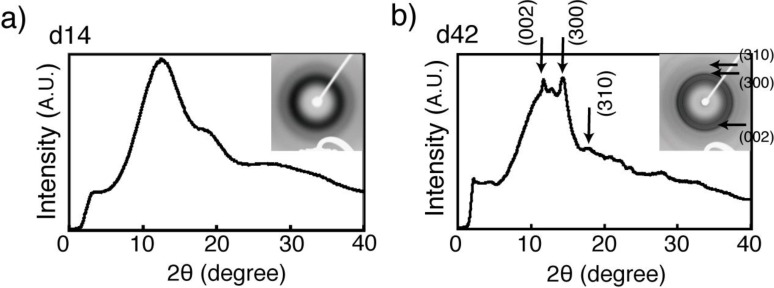
X-ray diffraction (XRD) profiles of obtained mineral in 3D construct at (**a**) day 14 and (**b**) at day 42.

**Figure 7:**
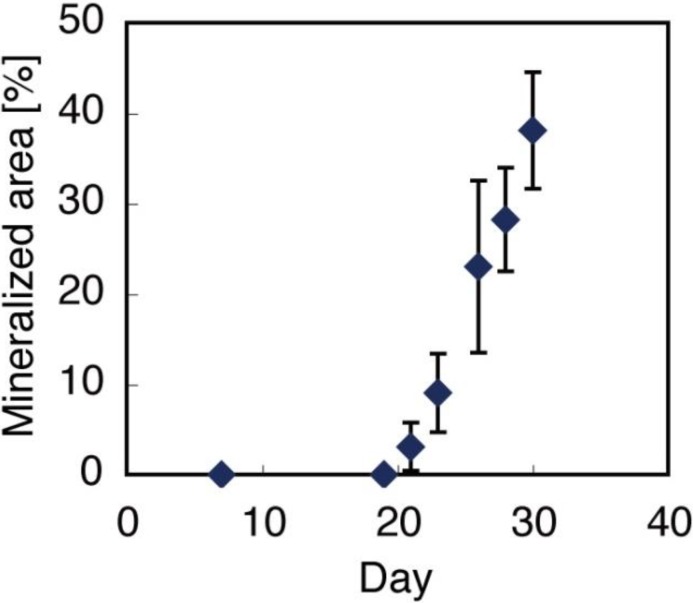
Calcification (nodule formation) in 2D bone marrow derived mesenchymal stem cells (BMSCs) culture was detected at day 19; the area increased in accordance with the culture period.

Von Kossa staining of the gel detected mineral deposition on day 35 of culture. The mineral deposition region was initially localized only at the cell surroundings; this extended to a wider region on day 42. Similar to the matrix deposition, the amount of mineral deposition increased in accordance with the cell culture period, *i.e*., the brown stains indicating minerals also darkened (Figure 4). SEM images also showed that the number of mineralized matrix vesicles increased in accordance with the culture period (Figure 5). An X-ray diffraction (XRD) profile of the obtained minerals in the 3D construct at day 42 showed the specific peaks of (002), (300), and (310) derived from hydroxyapatite (HAp), which were not detected in the constructs at day 14 (Figure 6). Thus, 3D gel material composed of cells, matrix proteins, and HAp was obtained by BMSCs cultured in 3D fibrin gel. Octaclcium phosphate (OCP) is generally considered to be the precursors of HAp in biologic hard tissue development [21,22], however, our results suggested that the microenvironment synthesized by cells and matrix with well-controlled pH might promote HAp formation instead of OCP formation. Similar to previous researches [23,24], nodule formation, *i.e*., mineral deposition, in a 2D BMSCs culture with an osteogenic differentiation medium was observed at day 19 (Figure 7). These results suggested that cell-derived mineralization in the 3D cell culture condition was delayed compared to that in a normal 2D culture condition. This was probably due to the low cell density used in this 3D cell culture compared to that in a normal 2D cell culture. The high cell density in the 3D culture causes cell apoptosis in the internal region of the gel because of hypoxia [25,26]. Therefore, further optimization of the cell culture conditions would be necessary to facilitate the osteogenic differentiation in this system.

### 2.3. Composition of Obtained 3D Constructs

**Figure 8:**
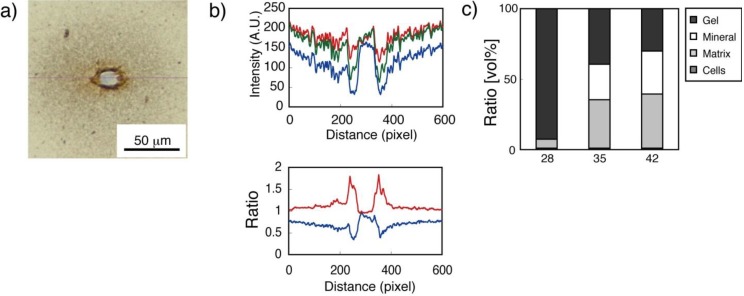
(**a**) Representative von Kossa-stained image of a single bone marrow derived mesenchymal stem cell (BMSC) in fibrin gel. Unstained cell nuclear, dark stained cell plasma, and distributed mineral deposition around the cell are observed. Horizontal line crossing center of cell nuclear used to obtain RGB color information; (**b**) Line profile of color intensity in each RGB color obtained from line shown in (a) (upper). Red line obtained by R/G from RGB color intensity indicates mineral deposition; blue line obtained by B/G from RGB color intensity indicates cell nuclear (lower); (**c**) Estimated volume ratio of components in 3D gel construct with different culture periods.

As shown in Figure 8, the von Kossa-stained section was divided into the following regions: unstained cell nuclear, darker stained cell matrix, and radial distribution of mineral deposition surrounding the cell. Based on both stained samples (HE and von Kossa), the volume composition of 3D constructs obtained with the different culturing periods was estimated. The calculated data showed that the fibrin gel volume decreased from 92% (day 28) to 30% (day 42), while the matrix protein increased from 6% (day 28) to 38% (day 42), and the minerals increased from 0 (day 28) to 31% (day 42) of the cell culture period. Thus, the composition in the construct altered from a fibrin-gel-rich phase (day 28) to a matrix-protein-rich phase (day 35). A mineral-rich phase in the construct was finally obtained at day 42.

### 2.4. Osteoconductivity of Synthesized Materials

**Figure 9:**
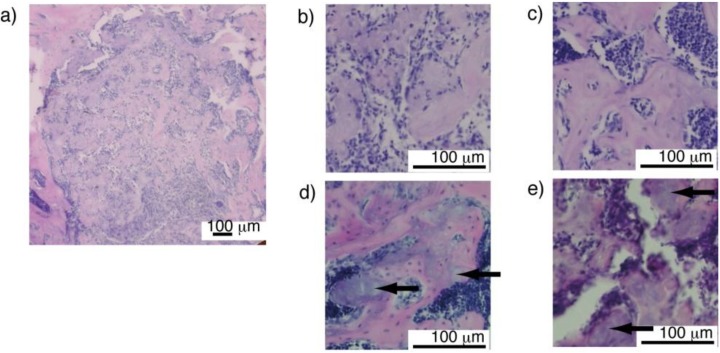
(**a**) Representative image of newly formed bone containing composite gel material in defect region of mouse tibia; (**b–e**) Implanted composite gel material was separated into several parts. Separated parts (colored purple within pink bone matrix) were surrounded by newly formed bone colored pink (b; day 14, c; day 28, d; day 35, e; day 42). Bone marrow cells penetrated into spaces in newly formed bone. Arrows indicate retained minerals in implanted gel material (d,e).

To evaluate the osteoconductivity of the obtained 3D constructs, the constructs from the 14–42 days of culture were implanted in artificially prepared bone defect regions in mice tibiae. Fibrin gel has been investigated as drug or cell vehicles for tissue engineering and other biomedical applications, and has shown great biocompatibility [27,28,29]. Our histological results indicated that all the implanted materials showed excellent biocompatibility and osteoconductivity. Moreover, each implanted sample was divided into several parts. Newly formed bone showed trabecular bone like structures (Figure 9). Bone marrow cells were observed in the space among the newly formed bone, indicating that the implanted material had potential for osteogenesis and sufficient biodegradability. Notably, minerals contained in the 3D constructs clearly remained in the newly formed trabecular bone matrix suggesting that the difference in composition of materials might be effective for controlling the degradation period of materials or the healing period for bone regeneration.

## 3. Experimental Section

### 3.1. BMSCs Culture in Fibrin Gel

A fibrinogen solution (4 mg/mL, Sigma Aldrich, MO, USA) containing aprotinin (5 mg/mL, Sigma Aldrich) was mixed with a thrombin solution (2.5 U/mL, Sigma Aldrich) in a 1:1 ratio. Subsequently, 400 μL of the resultant solution was poured into a silicone mold (length = 10 mm; diameter = 6 mm) and incubated (37 °C) for 20 min. For cell culture in fibrin gel, BMSCs (2.0 × 10^4^ or 8.0 × 10^4^ cells/mL) derived from bulb/c mouse bone marrow were added to the fibrinogen solution prior to gelation. The formed gel was removed from the mold and cultured in an osteogenic differentiation medium containing L-ascorbic acid, beta-glycerophosphate, dexamethason (Sigma Aldrich), and bone morphogenic protein-2 (BMP-2, Pepro Tech, NJ, USA) in a floating condition. Alteration of the gel size was characterized by optical images with image analysis software (Image J, NIH, MD, USA).

### 3.2. Deposition and Distribution of Matrix and Minerals in Fibrin Gel

To evaluate the deposition and distribution of the secreted matrix and precipitated minerals in the gel, section staining was conducted. The fibrin gels were fixed with 4% paraformaldehyde and embedded in paraffin. Sections (5 μm thick) were either stained with hematoxylin and aqueous eosin Y solution (Sigma Aldrich) to visualize the matrix deposition and distribution, or von Kossa staining was conducted to evaluate the deposition and distribution of minerals in the gel. For real time RT-PCR study, total RNA of osteoblasts in fibrin gel at different strain rates was extracted using Trizol reagent (Invitrogen, CA, USA), and transcribed to cDNA using oligo-dT and superscript reverse transcriptase (Invitrogen) according to the manufacturer’s instructions. mRNA expression of osteopontin (Opn), osteocalcin (Oc) and glyceraldehydes-3-phosphate dehydrogenase (Gapdh) were analyzed with a 7300 real time RT-PCR System (Applied Biosystems, CA, USA). Immunofluorescent staining of type I collagen (rabbit anti-mouse type I collagen; Chemicon, MA, USA) and nuclear staining with hoechst33342 (Invitrogen, CA, USA) were carried out to understand the localization of type I collagen in 3D gel.

Mineral deposition in the 3D fibrin gel was also observed using scanning electron microscopy (SEM, JSM-6390, JEOL, Japan). For the SEM observation, samples were fixed with 4% paraformaldehyde, rinsed with phosphate buffered saline (PBS), post-fixed with 1% OsO_4_, and rinsed with PBS. The gels were dried with a critical-point drier using CO_2_. The mounted sample was coated with gold. To observe the internal region in the 3D constructs, a freeze fracturing method using dimethyl sulfoxide (DMSO) was applied [30]. Precipitated matrix vesicles were evaluated by image analysis software (Image J, NIH, MD, USA). X-ray diffraction analysis (XRD) was carried out using a microbeam X-ray diffractometer system (M18XHF22-SR, Mac Science, Japan). Mo-Kα radiation was generated at a tube voltage of 40 kV and a tube current of 90 mA, and the incident beam was focused onto a beam spot 100 μm in diameter by a collimeter. As a result, diffraction of (002), (300), and (310) could be detected from each diffraction plane with a plane normal in-cone arrangement.

### 3.3. Image Analysis

To evaluate the alteration of the 3D construct compositions, a total of 20 stained images randomly picked from each group (n = 4) were used for the image analysis (Photoshop CS, Adobe, CA, USA). The following processes and definitions were applied to estimate the volume composition of the obtained 3D constructs.

(1) Color information expressed in RGB color was obtained from each stained image (HE or von Kossa).
(2) A mineral (X) in the imaged area was defined as the region where both blue/green < 0.85 and red/green >1.02 were acquired in the von Kossa-stained samples. A matrix (Y) was defined as the region where 210 < red < 225 was acquired in the HE-stained samples. A cell (Z) was fixed as 1.5% in volume because our previous work indicated that proliferation of myoblasts or osteoblasts in 3D fibrin gel slightly increased at the start of culture and saturated by day 5 and that the number of cells was maintained following the culture period [31].
(3) The volume ratio of each component was expressed as the following equation.
Vx = Mineral(X)/f, Vy = Matrix(Y)/f, Vz = 1.5/f
(1)
Fibrin gel = 100 × (1 − Vx − Vy − Vz)
(2)
f = {initial gel volume—gel volume (day 42)}/42
(3)

### 3.4. Osteoconductivity of Synthesized Materials

Treatment of the experimental animals was in accordance with the Osaka University Animal Care Guidelines, and the procedures were approved by the animal research committee in Osaka University (No.19-045-0). Balb/c mice (CLEA, Japan) were anesthetized by intraperitoneal injections of anesthetics (Pentobarbital, Dainihon Pharmacy, Japan). Prepared 3D constructs (14–42 days of culture) were implanted in bone defect sites formed in their right tibiae. Samples (n = 4) extracted 3 weeks after implantation were fixed for 24 h in 10% formaldehyde and were subsequently decalcified through a 5-day incubation in 10% formic acid. Thin section staining (HE staining) was carried out according to the methods mentioned above.

### 3.5. Statistical Analysis

All quantitative tests were carried out in quadruplicate, and mean values with standard deviations were calculated. The data was statistically analyzed by one factor analysis of variance (ANOVA). Student’s t-test was used for comparison at a 99% confidence interval.

## 4. Conclusions

Organic/inorganic composite gel material was successfully fabricated by culturing BMSCs in 3D fibrin gel matrices. In addition, the material composition was regulated by altering the cell culture period. This cell-based fabrication of composite material would be not only an effective method to obtain functional materials for bone tissue engineering purposes, but also an excellent tool to understand the biomineralization process *in vitro*.
